# Augmented ***β*-Cell Function and Mass in Glucocorticoid-Treated Rodents Are Associated with Increased Islet Ir-*β***/AKT/mTOR and Decreased AMPK/ACC and AS160 Signaling

**DOI:** 10.1155/2014/983453

**Published:** 2014-09-17

**Authors:** André O. P. Protzek, José M. Costa-Júnior, Luiz F. Rezende, Gustavo J. Santos, Tiago Gomes Araújo, Jean F. Vettorazzi, Fernanda Ortis, Everardo M. Carneiro, Alex Rafacho, Antonio C. Boschero

**Affiliations:** ^1^Department of Structural and Functional Biology, Institute of Biology, University of Campinas (UNICAMP), P.O. Box 6109, 13083-970 Campinas, SP, Brazil; ^2^School of Medical Sciences, University of Campinas (UNICAMP), Campinas, SP, Brazil; ^3^Department of Cell and Developmental Biology, Institute of Biomedical Science, University of São Paulo (USP), São Paulo, SP, Brazil; ^4^Department of Physiological Sciences, Center of Biological Sciences, Federal University of Santa Catarina (UFSC), Florianópolis, SC, Brazil

## Abstract

Glucocorticoid (GC) therapies may adversely cause insulin resistance (IR) that lead to a compensatory hyperinsulinemia due to insulin hypersecretion. The increased *β*-cell function is associated with increased insulin signaling that has the protein kinase B (AKT) substrate with 160 kDa (AS160) as an important downstream AKT effector. In muscle, both insulin and AMP-activated protein kinase (AMPK) signaling phosphorylate and inactivate AS160, which favors the glucose transporter (GLUT)-4 translocation to plasma membrane. Whether AS160 phosphorylation is modulated in islets from GC-treated subjects is unknown. For this, two animal models, Swiss mice and Wistar rats, were treated with dexamethasone (DEX) (1 mg/kg body weight) for 5 consecutive days. DEX treatment induced IR, hyperinsulinemia, and dyslipidemia in both species, but glucose intolerance and hyperglycemia only in rats. DEX treatment caused increased insulin secretion in response to glucose and augmented *β*-cell mass in both species that were associated with increased islet content and increased phosphorylation of the AS160 protein. Protein AKT phosphorylation, but not AMPK phosphorylation, was found significantly enhanced in islets from DEX-treated animals. We conclude that the augmented *β*-cell function developed in response to the GC-induced IR involves inhibition of the islet AS160 protein activity.

## 1. Introduction

Glucocorticoids (GCs), such as dexamethasone (DEX), are widely prescribed in clinical practice due to their anti-inflammatory, antiallergic, and immunosuppressive properties. GCs are the standard treatment for asthma, rheumatoid arthritis, systemic lupus erythematosus, and inflammatory bowel diseases [1, 2], as well as the protection against rejection of transplanted organ [1]. However, supraphysiological levels of GCs (either exogenous or endogenous) induce adverse effects related to glucose homeostasis, such as decreased peripheral insulin sensitivity, glucose intolerance, and dyslipidemia [3–6]. Depending on the genetic background, age, and time and dose of the exposure, it can also lead to type 2 diabetes mellitus (T2DM) [4, 6–11].

T2DM is a multifactorial metabolic disease mainly characterized by hyperglycemia [12], but before the occurrence of overt hyperglycemia, peripheral insulin resistance (IR) leads to compensatory insulin hypersecretion by pancreatic islets [13]. This adaptive islet compensation leads to a state of hyperinsulinemia together with normoglycemia or a modest increase in glycemic values (prediabetic state) that persist until the *β*-cells can handle the required demand for insulin [14]. The major mechanisms by which *β*-cells generate hyperinsulinemia during adaptive compensation consist of functional (e.g., increased insulin biosynthesis and/or secretion) and structural adaptations (e.g., increased *β*-cell hyperplasia and hypertrophy that may result in increased *β*-cell mass) [14–16]. Thus, when *β*-cells can no longer compensate, a glucolipotoxicity process progressively develops that induces *β*-cell death accompanied by hypoinsulinemia, hyperglycemia, and hyperlipidemia [15].

The *β*-cell compensations [17] can be rapidly obtained experimentally by 5-day treatment with DEX (5 days) [18, 19] that induces peripheral IR [20, 21], which is associated with increased hepatic gluconeogenesis and lipolysis [8].

Rats and mice are widely used as laboratory models to elucidate the mechanisms (at the functional, structural, and molecular levels) involved in the pancreatic islets compensations during the development of T2DM, such as observed in GC-induced IR [9, 10, 18–27]. These compensations are strongly associated with increased insulin signaling in islets (insulin receptor [Ir] and the protein kinase B [AKT]) [9, 19, 25, 28]. The AKT1 overexpression in *β*-cells lead to increased islet mass [29], while the *β*-cell-specific Ir knockout (*β*IrKO) lead to reduced glucose-stimulated insulin secretion (GSIS), lower islet insulin content, and glucose intolerance, supporting the consensus that the loss of insulin action on *β*-cells leads to *β*-cell failure and T2DM [30].

It has been demonstrated that a major downstream effector of AKT, the AKT substrate with 160 kDa (AS160), previously recognized as an important protein in insulin signaling in skeletal muscles and adipose tissue, is found to be expressed in *β*-cells and is also a major effector of AKT in the *β*-cell [31, 32].

In skeletal muscles, both insulin and AMP-activated protein kinase (AMPK) pathways phosphorylate and inhibit AS160 [32], inducing the trafficking of vesicles containing glucose transporter-4 (GLUT4) to the plasma membrane [33, 34]. In *β*-cells, AS160 plays an important role in the GSIS [31] and evidences suggest that AS160 is also involved in the trafficking of vesicles containing insulin to the plasma membrane [35]. AS160 is expressed in human islets and its phosphorylation is increased when stimulated with glucose (16.7 mmol/L) [31]. Also, T2DM humans display reduced AS160 mRNA and the AS160 knockdown in primary mouse islets lead to increased basal insulin secretion (2.8 mmol/L glucose), whereas GSIS (16.7 mmol/L glucose) is impaired [31].

Considering the importance that has been given to the involvement of the AS160 in islet function, we sought to investigate whether the AS160 content and phosphorylation could be modulated by the GC treatment. By using two experimental models, Swiss mice and Wistar rats, we demonstrated that the augmented *β*-cell function (insulin hypersecretion and increased *β*-cell proliferation) caused by 5-day DEX administration is associated with significant reduction of the AS160 protein content and marked increase of the AS160 protein phosphorylation in islets of both insulin-resistant rodents. Furthermore, this AS160 inhibition is islets from GC-treated animals which were accompanied by high AKT, but not AMPK phosphorylation.

## 2. Materials and Methods 

### 2.1. Reagents

Dexamethasone phosphate (Decadron) was purchased from Aché (Campinas, SP, Brazil). Human recombinant insulin (Humalin R) was obtained from Lilly (Indianapolis, IN, USA). Trizol was purchased from Gibco-BRL (Gaithersburg, MD, USA) and Triton X-100 was purchased from Cromato Products (Diadema, SP, BR). The ^125^I-labeled insulin used in the radioimmunoassay (RIA) was purchased from Perkin Elmer (Boston, MA, USA). SDS-PAGE and immunoblotting were performed using Bio-Rad systems (Hercules, CA, USA), and all chemicals were from Bio-Rad and Sigma Aldrich (St. Louis, MO, USA). Western blot detection of specific proteins used the following primary antibodies from Santa Cruz (Santa Cruz, CA, USA): anti-phospho-Ir-*β* ^Tyr1162/1163^, anti-Ir-*β*, anti-phospho-AKT_1/2/3_^Thr308^, anti-AKT_1/2/3_, anti-phospho-ERK^Tyr204^, anti-ERK_1_, anti-PKC, and anti-GAPDH. Anti-phospho-AMPK^Thr172^, anti-AMPK, anti-phospho-ACC^Ser79^, anti-ACC, anti-phospho-AS160^Thr642^, anti-phospho-PKC substrates, and anti-PCNA were from cell signaling (Temecula, CA, USA). Anti-phospho-mTOR^Ser2448^ and anti-mTOR were from Abcam (Cambridge, UK), and anti-CX36 was from Invitrogen (Camarillo, CA, USA). The secondary antibodies used were anti-rabbit IgG and anti-mouse IgG from cell signaling. Urea antiprotease/antiphosphatase buffer was composed of 7 M urea, 2 M thiourea, 5 mmol/L EDTA, 1 mmol/L sodium fluoride, 1 mmol/L sodium orthovanadate, 1 mmol/L sodium pyrophosphate, 2 mM PMSF, 1% Triton X-100, and 1 *μ*g/mL aprotinin (Trasylol from Bayer Health Care Pharmaceuticals, Berkley, CA). Immunohistochemical detection of insulin was performed using an anti-insulin primary antibody (guinea pig polyclonal) (Dako, Carpinteria, CA, USA) and detection of KI-67 was performed using an anti-KI-67 antibody (Spring Bioscience, Pleasanton, CA, USA). The secondary antibodies used to detect the anti-insulin and anti-KI-67 antibodies were anti-guinea pig IgG (Invitrogen, Carlsbad, CA, USA) and HRP-conjugated anti-rabbit IgG (Nichirei Bioscience, Tokyo, JP), respectively.

### 2.2. Animals and Experimental Design

Experiments were performed on groups of male Swiss mice and male Wistar rats (80 to 100 days) obtained from the State University of Campinas Animal Breeding Center. They were maintained in appropriate animal cages and kept at 24°C on a 12 : 12-hour light-dark cycle. Both mice and rats had access to food and water ad libitum. The experiments with animals were approved by the Institutional State University of Campinas Committee for Ethics in Animal Experimentation under protocol number 2285-1. Mice and rats were divided into the following two groups: DEX-treated rodents (DEX) that received a daily injection of dexamethasone phosphate (intraperitoneally (i.p.), 1.0 mg/kg body weight (b.w.) in 0.9% NaCl for 5 consecutive days) and control rodents (CTL) that received a daily injection of saline (i.p., 1.0 mL/kg b.w., for 5 consecutive days) between 08:00 and 09:00. All experiments were performed 24 h after the last DEX injection (at the sixth day) to avoid the overlapping of acute and chronic effects of GCs.

### 2.3. Metabolic, Hormonal, and Biochemical Measurements

Body weight was measured 2 days before the start of treatment and each day thereafter until the day of euthanasia. On the day after the last DEX administration, blood was collected from the tails of a group of fasted (10–12 h) animals and blood glucose levels were measured with a glucometer (Accu-Chek Advantage, Roche Diagnostic, Switzerland). Immediately after blood glucose determination, the animals were sacrificed (by exposure to CO_2_ followed by decapitation) and the trunk blood was collected. The serum was obtained by centrifugation and was used to measure the following parameters: insulin by RIA, nonesterified-free-fatty-acids (NEFA) (Wako Chemicals; Richmont, USA), triacylglycerol (TG), and total cholesterol (CHOL) (Roche/Hitachi; Indianapolis, IN, USA) by spectrophotometer according to the manufacturers' instructions.

### 2.4. Intraperitoneal Insulin Tolerance Test (ipITT)

A separate group of fed animals received an intraperitoneal injection of insulin (1 U/kg b.w. in 0.9% NaCl). Blood glucose was measured at baseline (before insulin administration; 0 min) and at 5, 10, 15, 30, 45, and 60 min after insulin administration. Blood glucose measurements were then converted into the natural logarithm (Ln); the slope was calculated using linear regression (time × Ln[glucose]) and multiplied by 100 to obtain the constant rate of glucose decay per minute (%/minute) during the ipITT (KITT) [12].

### 2.5. Intraperitoneal Pyruvate Tolerance Test (ipPTT)

A separate group of fasted (14 h) animals received an i.p. injection of pyruvate (1 g/kg b.w.). Blood glucose was measured at baseline (before pyruvate administration; 0 min) and at 5, 15, 30, and 60 min after pyruvate administration. The area-under-curve (A.U.C.) for blood glucose values was obtained from the 30 min of the ipPTT after normalization of the data [10]. The constant rate of glucose appearance per minute (%/min) during the first 15 min of the ipPTT (KPTT) was calculated as described above.

### 2.6. Intraperitoneal Glucose Tolerance Test (ipGTT)

A separate group of fasted (10 h) animals received an i.p. injection of 25% glucose solution (1 g/kg b.w.). Blood glucose was measured at baseline (before glucose administration; 0 min) and at 15, 30, 60, 90, and 120 min after glucose administration. The A.U.C. was calculated as described above.

### 2.7. Islet Isolation and Static Insulin Secretion

Islets were isolated by collagenase digestion of the pancreas as described [27]. Insulin secretion and quantification by RIA were performed using a similar method as described previously [10].

### 2.8. Immunohistochemistry and Morphometry in the Endocrine Pancreas

For morphometric analysis, at least 6 pancreases from each group of mice and rats were removed, weighed, and fixed for 24 hours in 4% paraformaldehyde solution, as previously described [25]. For morphometry analysis, all islets present in the sections were obtained systematically by capturing images with a digital camera (Olympus DP52, Tokyo, JP) coupled to a microscope (Olympus BX51TF, Tokyo, JP). The islet, *β*-cell and section areas were analyzed using the free software ImageJ (http://rsbweb.nih.gov/ij/download.html). The relative *β*-cell area was calculated by dividing the *β*-cell area per section by the total pancreas area per section and the absolute *β*-cell mass was calculated by multiplying the pancreas weight by the relative *β*-cell area per pancreas. The relative number of islets was obtained by dividing the number of islets per section by the total area of the section [25].

### 2.9. *β*-Cell Proliferation

Average *β*-cell proliferation was obtained by counting the total islet cell nuclei stained for insulin and KI-67 using the same software cited above. *β*-cell proliferation was estimated by dividing the number of KI-67-positive nuclei by the total number of insulin-positive cells [25].

### 2.10. *β*-Cell Death

Fragmented DNA was isolated using buffer *A* containing 50 nM Tris-Hcl pH 8.1, 10 nM EDTA, and 1% Triton X100. Total DNA was isolated using buffer *B* containing 50 nM Tris-Hcl pH 8.1, 10 nM EDTA, and 1% SDS. After isolation, total DNA and fragmented DNA were precipitated with phenol, chloroform and isoamyl alcohol (25 : 24 : 1) and quantified by SybrGreen fluorescence using a standard curve (0.5 ng/mL to 50 ng/mL). The data are expressed as the ratio of fragmented to total DNA.

### 2.11. Protein Extraction and Immunoblotting

Protein extraction and immunoblotting were performed as previously reported [36] with minor modifications. Images were captured by the luminescent image analyzer LAS-3000 (Fujifilm, Tokyo, JP) and the specific band intensity was quantified by optical densitometry using ImageJ.

### 2.12. RNA Isolation and Quantitative RT-PCR Analysis

Groups of 600 islets were homogenized in Trizol following phenol chloroform RNA extraction, as previously described [36]. Relative quantities of target transcripts were calculated from duplicate samples after normalization of the data against the endogenous control, GAPDH. The primers used were as follows: PDX1 (S: aaccggaggagaataagagg and AS: gttgtcccgctactactgtt), insulin (S: ttgcagtagttctccagtt and AS: attgttccaacatggccctgt), and GAPDH (S: cctgcaccaccaactgctta and AS: gccccacggccatcacgcca).

### 2.13. Statistical Analysis

The results are expressed as the mean ± s.e.m. of the indicated number (*n*) of animals. A paired or unpaired Student's *t*-test was used for intragroup (before and after) or intergroup (CTL versus DEX group) comparisons. All analyses were performed using GraphPad Prism version 5.0 (GraphPad Software, San Diego, CA, USA). A *P* value less than or equal to 0.05 was considered significant.

## 3. Results

### 3.1. DEX Treatment Reduced Body and Adrenal Gland Weights in Mice and Rats

It is known that 5-day DEX treatment in rats produces a dose-dependent reduction in adrenal gland mass in a reciprocal reduction of endogenous corticosterone concentration [10]. As expected, DEX treatment induced a significant decrease in the mass of the adrenal glands in mice (30%) and rats (35%) compared with their respective controls (Table 1), which demonstrates the effectiveness of exogenous GC treatment on adrenal hypotrophy. In addition, the mice and rats showed reduced body weight (4% and 11%, resp.) (Table 1), which is a feature commonly observed in rats made insulin-resistant by DEX treatment [10, 37].

### 3.2. DEX Treatment Induced a Reduction in Insulin Sensitivity in Mice and in Rats but Increased Hepatic Gluconeogenesis and Glucose Intolerance Only in Rats

**Figure 1:**
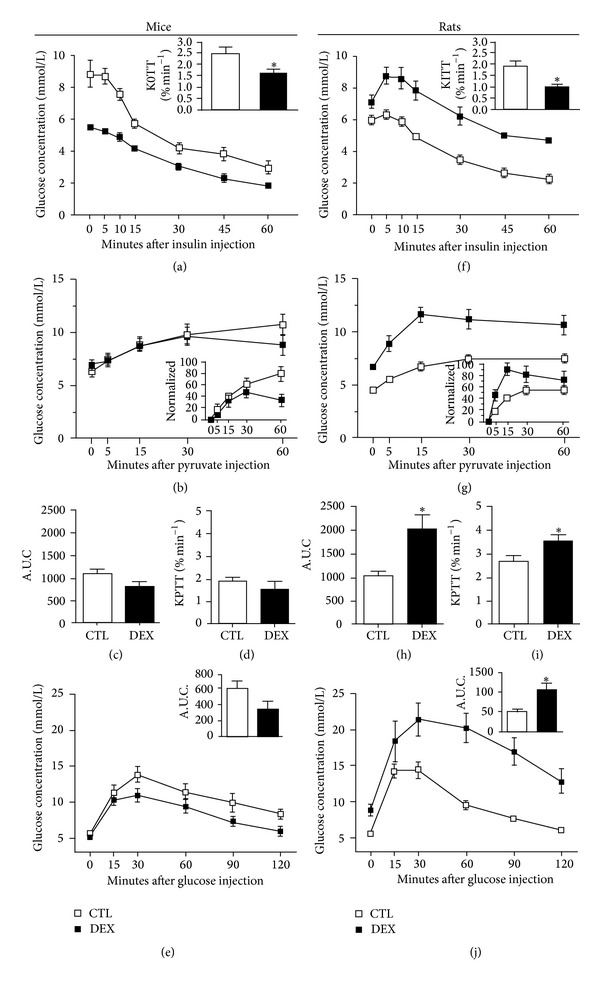
DEX treatment induces a reduction in insulin sensitivity in mice and in rats but increases hepatic gluconeogenesis and glucose intolerance only in rats. ((a), (f)) Blood glucose during intraperitoneal insulin tolerance test (ipITT; 1 U/Kg b.w.) in DEX-treated mice and rats, respectively; the inset in (a) and (f) depicts the constant rate of glucose disappearance (KITT). ((b), (g)) Intraperitoneal pyruvate tolerance test (ipPTT; 1 g/Kg b.w.) in DEX-treated mice and rats, respectively; the inset in (b) and (g) depicts the ipPTT data normalized by minute 0 ((c), (h)) A.U.C and ((d), (i)) the constant rate of glucose appearance (KPTT) during ipPTT in DEX-treated mice and rats, respectively. ((e), (j)) intraperitoneal glucose tolerance test (ipGTT; 1 g/Kg b.w.) in DEX-treated mice and rats, respectively; the inset in (e) and (j) depicts the A.U.C. from ipGTT; values are mean ± S.E.M.; *n* = 8–10 animals per group. *Significantly different compared to CTL. Unpaired Student's *t*-test, *P* ≤ 0.05.

We first confirmed the reduction in insulin sensitivity in both mice and rats. The ipITT revealed a significant reduction in insulin sensitivity in both DEX-treated groups (Figures 1(a) and 1(f), resp.) as indicated by the reduction in the KITT (inset in Figures 1(a) and 1(f)), although this effect occurred to a lesser extent in mice. We also analyzed whether GC treatment increased hepatic gluconeogenesis. DEX treatment did not alter gluconeogenesis in mice; however, DEX-treated rats showed increased glucose production in response to pyruvate administration, as indicated by the increased A.U.C. and the KPTT (Figures 1(b)–1(d) and 1(g)–1(i), resp.), which indicates hepatic insulin resistance. Despite a reduction in insulin sensitivity, DEX-treated mice remain glucose tolerant (Figure 1(e)). Compared to their controls, DEX-treated rats showed the well-known negative impact of GC excess on glucose tolerance (Figure 1(j)), which reflects the association of increased hepatic glucose production with a possible reduction of peripheral glucose disposal.

### 3.3. DEX Treatment Induced Dyslipidemia and Hyperinsulinemia in Both Mice and Rats

DEX treatment increased fasting serum cholesterol (CHOL) and triacylglycerol (TG) concentrations in both mice and rats compared to their respective controls, which indicates a negative impact of GCs on lipid metabolism in both species. In addition, DEX treatment increased the NEFA levels only in rats (Table 2), which may indicate an increased rate of lipolysis of adipose tissue. DEX-treated rodents also showed a marked increase in serum insulin levels that were 1- and 9-fold higher in mice and rats, respectively. Blood glucose was not altered in DEX-treated mice, while it was 60% higher in DEX rats compared to CTL (Table 2). Thus, the hyperinsulinemia corroborates the IR state and seems to protect against the disruption of glucose homeostasis, though GC-treated rats were glucose intolerant.

### 3.4. DEX Treatment Increased the Responsiveness to Glucose in Islets from Both Mice and Rats

**Figure 2:**
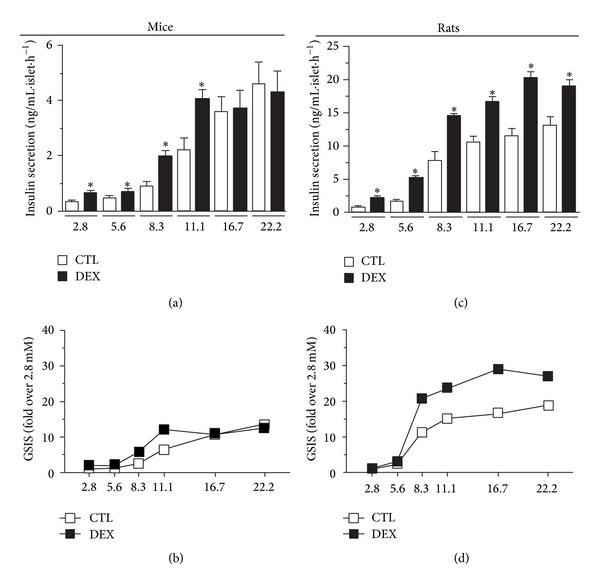
Islets from DEX-treated rats are more responsive to glucose than islets from DEX-treated mice. ((a), (c)) Static cumulative insulin secretion in isolated islets from DEX-treated mice and rats in response to different glucose concentrations, respectively. ((b), (d)) Normalized glucose-stimulated insulin secretion (GSIS) (fold increase in relation to 2.8 mmol/L glucose) in mice and in rats, respectively. Values are mean ± S.E.M.; *n* = 4–6 wells from 5 different animals.  *Significantly different compared to CTL. Unpaired Student's *t*-test, *P* ≤ 0.05.

Due to the increased insulinemia that was observed in both DEX-treated mice and rats, we assessed the GSIS. Compared to the control groups, isolated islets from DEX-treated rats were more responsive to all glucose concentrations used (from 2.8 mmol/L to 22.2 mmol/L), (Figures 2(c) and 2(d)), whereas islets from DEX-treated mice were more responsive to glucose up to a concentration of 11.1 mmol/L (Figures 2(a) and 2(b)). These data point to species differences in the increase of *β*-cell function that contributes to the different degrees of hyperinsulinemia found in each species.

### 3.5. DEX Treatment Leads to Increased *β*-Cell Mass in the Pancreas of Mice and Rats

**Figure 3:**
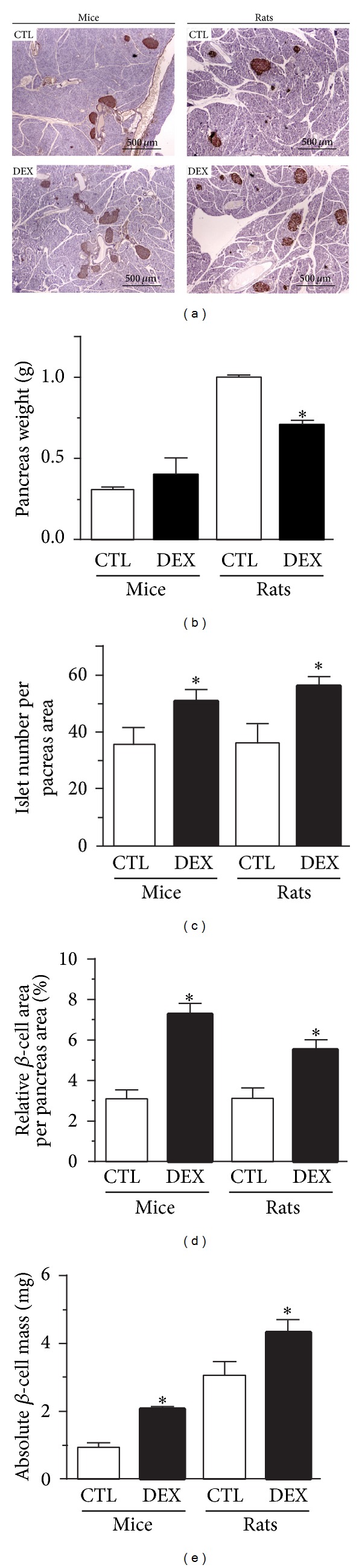
DEX treatment increases *β*-cell mass in the pancreas of mice and rats. (a) Representative pancreas sections stained for insulin with Hematoxylin counterstaining. (b) Pancreas weight, (c) relative islet number per pancreas area, (d) relative *β*-cell area per pancreas area, and (e) absolute *β*-cell mass in DEX-treated mice and rats. Values are mean ± S.E.M.; *n* = 5-6 animals per group (≈150 islets from mice and ≈300 islets from rats).  *Significantly different compared to CTL. Unpaired Student's *t*-test, *P* ≤ 0.05.

Because an increase in *β*-cell mass may also favor compensatory hyperinsulinemia, we investigated this parameter in DEX-treated mice and rats. Pancreatic sections stained for insulin revealed a significant increase in the number of islets per pancreatic area in both DEX-treated rats and mice compared to their respective controls (Figures 3(a) and 3(c)). Additionally, DEX treatment significantly increased the absolute *β*-cell mass in the pancreas from both mice and rats (Figure 3(e)). These data indicate a compensatory structural islet adaptation in response to DEX-induced IR in both species.

### 3.6. DEX Treatment Increases *β*-Cell Proliferation without Affecting Apoptosis in Mouse and in Rat Islet Cells

**Figure 4:**
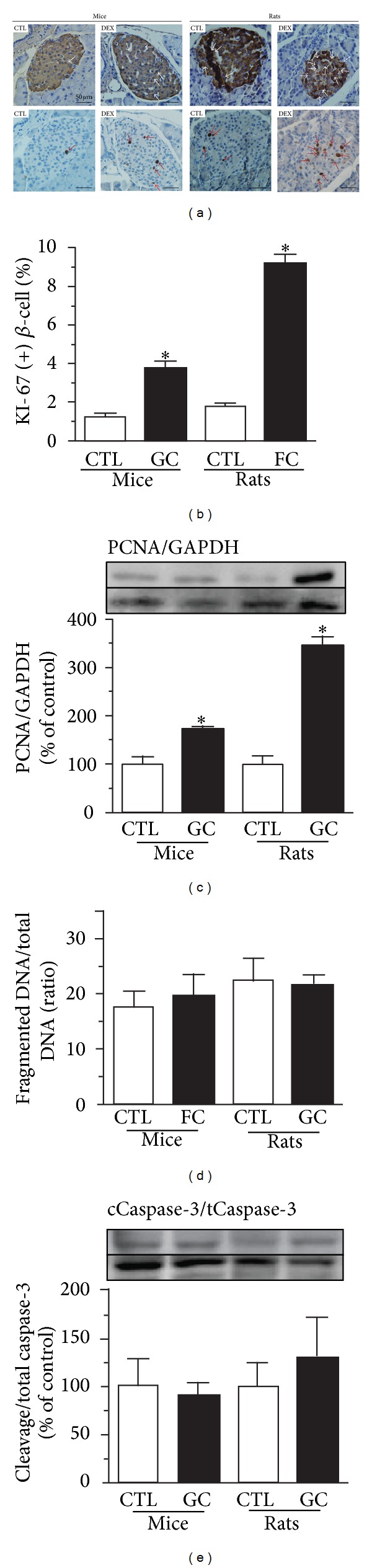
DEX treatment increases *β*-cell proliferation without affecting apoptosis in the islets of mice and rats. (a) Representative pancreas sections stained for insulin (on the top) and KI-67 (at bottom), (b) percentage of KI-67-positive nuclei (+) *β*-cell, (c) PCNA content, (d) DNA fragmentation/total DNA (ratio), and (e) caspase-3 cleavage/total caspase-3 ratio in islets from DEX-treated mice and rats. Values are mean ± S.E.M.; *n* = 5-6 rodents per group; ≈100 islets per species (≈8500 nuclei per group).  *Significantly different compared to CTL. Unpaired Student's *t*-test, *P* ≤ 0.05.

The *β*-cell mass is the result of a dynamic balance between cell death and proliferation. We found that DEX treatment significantly increased *β*-cell proliferation in islets from rats (420%) and mice (200%) compared with their respective controls, as indicated by the higher number of KI-67-positive *β*-cell nuclei (Figures 4(a) and 4(b)). In addition, DEX treatment increased the protein content of the proliferating cell nuclear antigen (PCNA) in islets to a greater extent in rats than in mice (Figure 4(c)). DEX treatment did not affect apoptosis in the islets of rats or mice as judged by DNA fragmentation (Figure 4(d)) and caspase-3 cleavage data (Figure 4(e)).

### 3.7. Increased *β*-Cell Function and Mass Is Associated with Increased Ir-*β*/AKT and Reduced AMPK/ACC Pathway Activities in Pancreatic Islets from DEX-Treated Mice and Rats

**Figure 5:**
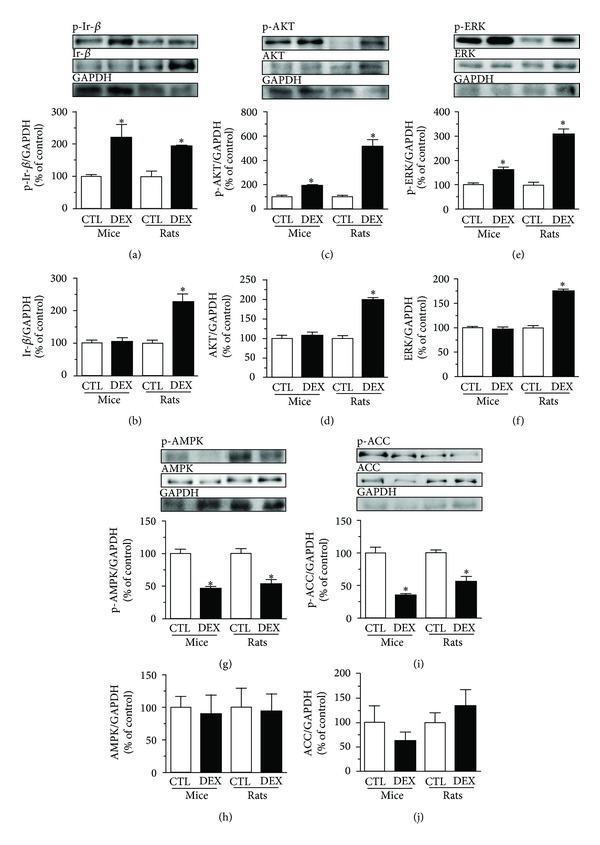
DEX treatment stimulates the canonical insulin pathway and inhibits the noncanonical insulin pathway in pancreatic islets from mice and rats. (a) Representative immunoblotting of phosphorylated and (b) total Ir-*β* content. (c) Phosphorylated and (d) total AKT content. (e) Phosphorylated and (f) total ERK content. (g) Phosphorylated and (h) total AMPK content. (i) Phosphorylated and (j) total ACC content in islets from DEX-treated mice and rats. Values are mean ± S.E.M.; *n* = 4 rodents per group.  *Significantly different compared to CTL. Unpaired Student's *t*-test, *P* ≤ 0.05.

Because insulin plays an important role in insulin secretion and in *β*-cell proliferation and can act directly upon the islet cells, we investigated whether insulin signaling was modulated by DEX treatment. We analyzed the canonical insulin pathway through the insulin receptor *β*-subunit (Ir-*β*) and its downstream protein, AKT. In islets from DEX-treated mice, we observed an increase in p-Ir-*β* (Figure 5(a)) without alterations in the total Ir-*β* protein content (Figure 5(b)). In islets from DEX-treated rats, the levels of p-Ir-*β* and total Ir-*β* protein increased significantly (Figures 5(a) and 5(b)). DEX-treated mice had increased islet p-AKT (Figure 5(c)) without alteration of the total AKT content (Figure 5(d)). In rat islets, DEX treatment resulted in higher amounts of p-AKT and total AKT (Figures 5(c) and 5(d)). We also assessed whether the extracellular signal-regulated kinase (ERK) pathway, which can be activated by insulin and participates in cell proliferation and differentiation [38], is modulated by DEX treatment. In mice islets, only p-ERK was augmented (Figures 5(e) and 5(f)), while DEX-treated rat islets showed a significant increase in p-ERK and total ERK content (Figures 5(e) and 5(f)). The AMPK pathway can also modulate the insulin secretion [39]. Islets from DEX-treated mice and rats had lower p-AMPK without alterations in the total AMPK content (Figures 5(g) and 5(h)). ACC, a downstream AMPK target protein, has diminished activity when phosphorylated. In both species, DEX treatment was associated with decreased levels of phosphorylated ACC (Figure 5(i)) without altering the total ACC levels in islets (Figure 5(j)), which indicates increased lipid synthesis in islets. Thus, DEX treatment results in increased insulin and decreased AMPK signaling pathways in islets from mice and rats.

### 3.8. DEX Treatment Modulates Proteins Related to Vesicle Trafficking, Protein Synthesis, Cell Growth, and Insulin Secretion in Pancreatic Islets from Both Mice and Rats

**Figure 6:**
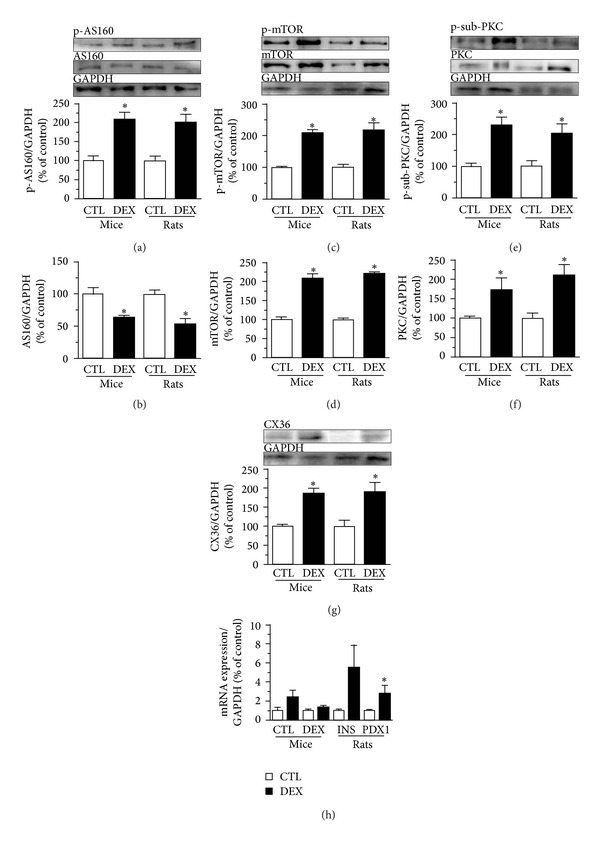
DEX treatment modulates proteins related to vesicle trafficking, protein synthesis, cell growth, and insulin secretion in pancreatic islets from both mice and rats. (a) Representative immunoblotting of phosphorylated and (b) total AS160 content. (c) Phosphorylated and (d) total mTOR content. (e) Phosphorylated substrates of PKC (range from 70 to 110 kDa) and (f) total PKC content. (g) CX36 expression and (h) mRNA expression of INS gene and PDX1 in islets from DEX-mice and rats. Values are mean ± S.E.M.; *n* = 4 rodents per group.  *Significantly different compared to CTL. Unpaired Student's *t*-test, *P* ≤ 0.05.

AKT and AMPK pathways were modulated in islets from GC-treated rodents. Since, in skeletal muscle, these pathways interact and inhibit AS160 through phosphorylation, allowing the GLUT4 vesicle trafficking to the plasma membrane [34], we investigated whether AS160 could be modulated in islets from GC-treated rodents. In mice and rats, DEX treatment resulted in higher p-AS160 (Figure 6(a)) and lower total AS160 contents (Figure 6(b)) compared to the control groups, which is in accordance with increased GSIS and hyperinsulinemia. AKT and AMPK pathway can also modulate the mammalian target of rapamycin (mTOR), a key protein that induces protein synthesis. Islets from DEX-treated mice and rats showed increased p-mTOR and total mTOR protein (Figures 6(c) and 6(d)).

Due to its importance in the insulin secretion process, we also investigated proteins related to calcium (Ca^2+^) influx, such as protein kinase C (PKC) and connexin 36 (CX36). The levels of phosphorylated PKC-target proteins (Figure 6(e)), total PKC (Figure 6(f)), and CX36 (Figure 6(g)) were increased in islets from DEX-treated mice and rats compared to their control groups. These data support an increased insulin secretion and *β*-cell mass in DEX-treated rodents.

### 3.9. DEX Treatment Is Associated with Increased mRNA Levels of PDX1 in Rat Islets, but Not in Mice Islets

We also evaluated whether DEX treatment could alter the mRNA levels of the insulin and pancreatic duodenal homeobox-1 (PDX1) genes. DEX treatment tended to increase insulin mRNA levels in pancreatic islets from both mice and rats (Figure 6(h); *P* = 0.056 and 0.055, resp.). In addition, DEX treatment increased PDX1 mRNA levels in rat islets, but not in mouse islets (Figure 6(h)).

## 4. Discussion

**Figure 7:**
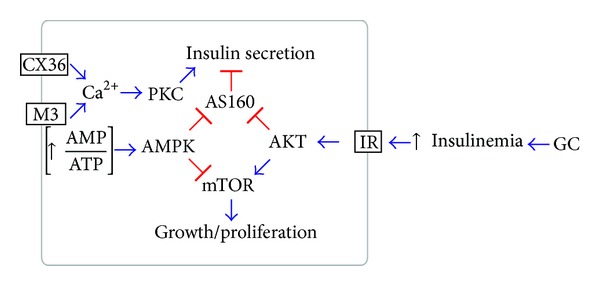
Hypothetical scheme of the main signaling pathways modulated in pancreatic *β*-cells by the* in vivo* glucocorticoid treatment. Decreased insulin sensitivity in peripheral tissues leads to hyperinsulinemia, which stimulates the insulin signaling in the pancreatic *β*-cell. Activated AKT inactivates the AS160, releasing its inhibitory effect on insulin secretion (functional compensation). Also, pAKT activates mTOR, favoring *β*-cell growth (structural compensation). In parallel, dephosphorylated AMPK, which may reflect a low AMP : ATP ratio, favors the activation of mTOR by AKT and suggest that AS160 inhibition is modulated by the insulin signaling, rather than AMPK signaling. An increased parasympathetic drive through muscarinic receptor (M3) and/or increased *β*-cells communication, through CX36 channels, may increase cytosolic Ca^2+^ and activates PKC, which stimulates insulin secretion. → represents stimulations and ⊣ represents inhibition.

In this study, we showed that* in vivo* GC treatment induced IR, hyperinsulinemia, and dyslipidemia in both species, but glucose intolerance and hyperglycemia only in rats. Both species displayed increased *β*-cell function (insulin hypersecretion) and mass (*β*-cell hyperplasia) as compensatory mechanisms. These compensatory responses were associated with reduced AS160 protein content and increased AS160 phosphorylation in islets as well as augmented AKT, but not AMPK phosphorylation (Figure 7).

The alterations in insulin action as well as in plasma insulin concentrations induced by the GC excess are compensated by enhanced insulin secretion in response to glucose, as can be frequently observed in GC-treated subjects [40–43]. Despite the pronounced hyperinsulinemia that results from a marked enhancement of the GSIS and increased *β*-cell mass, DEX-treated rats were not able to properly counteract the peripheral IR and both glucose intolerance and hyperglycemia developed being in accordance with previous studies [9, 19, 22, 37].

Although DEX-treated mice presented an increase in peripheral IR, they remained normoglycemic and glucose tolerant, indicating that the islet compensations (e.g., increased GSIS and *β*-cell mass) were sufficient to prevent any significant disruption of glucose homeostasis. Thus, at similar conditions of treatment (time and doses of DEX), mice seem to be less vulnerable than rats to the deleterious effects of GCs upon glucose homeostasis. This difference between species may be ascribed to difference in the GC receptor expression or activity in GC responsive tissues and/or in the GC metabolism, which does not exclude that for a more prolonged period and/or higher GC concentrations mice could develop an imbalance of the glucose homeostasis. These species-specific responses to the GCs regarding glucose tolerance highlight the importance to perform individualized analysis in patients receiving GC-based therapies.

Basal hyperinsulinemia may be explained by several factors, including the reduction of hepatic insulin clearance [44] and/or an increase of basal insulin secretion [10, 18]. The data obtained regarding insulin secretion demonstrated that both DEX-treated rats and mice had higher insulin responses to glucose, including at subthreshold glucose levels (e.g., 2.8 mmol/L and 5.6 mmol/L). It is well known that hydrocortisone administration acutely suppresses insulin release in mice by a mechanism that most likely involves the central activation of sympathetic nerves [45]. This was not the case here, as our experiments were performed 24 h after a 5-day course of DEX, thus eliminating the possible overlapping of acute and chronic effects.

Basal hyperinsulinemia in DEX-treated rodents can also be explained by the increased *β*-cell mass which is usually observed in insulin-resistant rodents fed a high-fat [17] or high sucrose diet [46]. Herein, we showed that increased *β*-cell mass resulted from, at least in part, increased *β*-cell proliferation as judged by the increased PCNA and KI-67 expression.

*β*-cell function that includes an increase of insulin response to glucose and increased *β*-cell proliferation are modulated by several stimuli through various intracellular pathways, and one of these major stimuli is insulin itself. Previous studies have documented the important role of insulin signaling in the *β*-cell function, survival, and growth [29, 30]. In our study, the compensatory insulin hypersecretion and *β*-cell proliferation in both DEX-treated rodents were associated with increased p-Ir-*β* and p-AKT in the islets, corroborating the hypothesis that enhanced insulin pathway (Ir-*β*/AKT) signaling is a common mechanism involved in the endocrine pancreas compensation, for example, after exposition to GCs (Figure 7). A previous study elegantly demonstrated that AKT overexpression sustains an elevation of the *β*-cell function that includes an enhancement of both GSIS and *β*-cell mass [29]. Our data with islet AKT protein content and phosphorylation are in accordance with other GC-related studies [19, 28]. Increased p-ERK also reinforces the hypothesis that insulin is involved with the pancreatic islet compensations during GC exposures. In DEX-treated rats, the increased Ir-*β*, AKT, and ERK total contents indicate an additive pancreatic compensatory mechanism compared to mice, in which the mechanism is limited to increased phosphorylation of the aforementioned proteins.

We also investigated the potential of AMPK signaling for mediation of islet adaptation in this context of GC excess. In muscle, AMPK is an energy-sensitizing enzyme that is active at low cellular energy (increased AMP/ATP ratio) [47]. In the *β*-cell lineage (MIN6), AMPK inhibition by glucose is essential for the activation of the insulin secretion process [39]. The decreased AMPK phosphorylation in islets from both groups of DEX-treated rodents supports the increased GSIS and may reflect the abundance of energy substrates available in the plasma. AMPK downregulation also underlies the increase in ACC activity and also suggests higher energy availability. Thus, we hypothesized that reduction in the AMPK/ACC pathway is one of the important mechanisms involved in the modulation of the altered insulin secretory process found under GC treatment.

In muscle and adipose cells, the AS160 is recognized as an inhibitor of GLUT4 vesicle trafficking to the plasma membrane [34]. In muscle cells, both insulin and AMPK signaling inhibit AS160 through phosphorylation, favoring GLUT4 vesicle trafficking [34]. *β*-cells also express AS160 that seems to exert several actions on the GSIS, survival and growth; and is under the control of insulin signaling [31, 35]. The crosstalk between AMPK signaling with the AS160 in islets is not yet well established. The increased AS160 phosphorylation in islets from GC-treated rodents indicates that insulin pathway, through the AKT, rather than the AMPK pathway, mediates AS160 inhibition, thus favoring the exocytosis of the insulin vesicles. In addition, the reduction in the AS160 expression is another mechanism that may contribute to increased insulin secretion (Figure 7).

The AKT and AMPK signaling also modulate mTOR function, a kinase that integrates multiple cell signals [13, 48] and regulates *β*-cell function and growth [48]. Our results agree with those from previous studies [49, 50] and indicate that the inhibition of AMPK and the activation of AKT synergistically activate mTOR in islets from GC-treated mice and rats, which may corroborate the increased GSIS and *β*-cell mass.

Another mechanism that contributes to the increased islet function after exposition to GCs may remain at the improvement of Ca^2+^ handling in *β*-cells. A Ca^2+^ influx contributes to the first (triggering) and second phase (amplifying) of insulin secretion [51]. An additional increase in intracellular Ca^2+^, under stimulatory glucose concentrations, is associated with a higher GSIS in islets from GC-treated rats [18]. The higher GSIS is also associated with increased CX36 expression in islets [27], which synchronizes Ca^2+^ transit between *β*-cells across the islets [52]. Activation of PKC, which is stimulated by Ca^2+^ and participates in the amplification of insulin secretion, is another mechanism associated with increased GSIS in rats treated with GCs [18]. Here, we also observed an increase in CX36 expression and an indirect increase in PKC activity in islets from both rats and mice treated with GCs, indicating the participation of Ca^2+^ in increased *β*-cell function. Finally, increased expression of *β*-cell markers (PDX1 mRNA) only in DEX-treated rats islets indicates that pancreatic compensations in this species also involve modifications at transcriptional levels.

## 5. Conclusions

We conclude that* in vivo* GC treatment induced in rodents, rats and mice, the deleterious effect on glucose homeostasis as observed in humans (IR, hyperinsulinemia, and dyslipidemia), which were compensated by the increased GSIS and *β*-cell mass. These compensations were associated with islet upregulation of AKT, but not AMPK signals that parallel with inhibited AS160 activation (Figure 7). We suggest that basal hyperinsulinemia in GC treated subjects may also involve the inhibition of AS160 in *β*-cells.

## Figures and Tables

**Table 1:** Body and adrenal weight in DEX-treated mice and rats.

	Mice			Rats		
	Body weight (g)		Adrenal weight (mg/100 g b.w.)	Body weight (g)		Adrenal weight (mg/100 g b.w.)
	Before	After	After	Before	After	After
CTL	38.7 ± 0.6	40.2 ± 0.6	18.21 ± 1.74	340.1 ± 6.5	351.2 ± 11.5	17.5 ± 1.12
DEX	39.4 ± 0.8	37.9 ± 0.7^†^	12.68 ± 0.72∗	346.7 ± 11.2	309.6 ± 10.8^†^	11.38 ± 0.78∗

^†^Significantly different using unpaired *t*-test versus DEX before treatment; *significantly different using unpaired *t*-test versus CTL after treatment *P* < 0.05; *n* = 6–8; values are mean ± s.e.m.

**Table 2:** Metabolic variables in 12 hour fasting DEX-treated mice and rats.

	Mice		Rats	
	CTL	DEX	CTL	DEX
Cholesterol (mg/dL)	169.7 ± 14.2	302.0 ± 18.3∗	23.8 ± 1.8	30.6 ± 1.7∗
Triacylglycerol (mg/dL)	106.9 ± 12.4	201.0 ± 13.8∗	111.2 ± 14.2	210.1 ± 26.6∗
NEFA (mMol/L)	1.2 ± 0.09	1.1 ± 0.08	0.6 ± 0.04	0.9 ± 0.09∗
Glycemia (mMol/L)	5.5 ± 0.3	5.0 ± 0.4	5.5 ± 0.08	8.8 ± 0.8∗
Insulinemia (pMol/L)	31.9 ± 3.09	59.9 ± 9.6∗	268.6 ± 64.3	2697.4 ± 439.0∗

*Significantly different using unpaired *t*-test versus CTL *P* < 0.05; *n* = 8–10; values are mean ± s.e.m.
